# Non-cell autonomous cues for enhanced functionality of human embryonic stem cell-derived cardiomyocytes via maturation of sarcolemmal and mitochondrial K_ATP_ channels

**DOI:** 10.1038/srep34154

**Published:** 2016-09-28

**Authors:** Wendy Keung, Lihuan Ren, Sen Li, Andy On-Tik Wong, Anant Chopra, Chi-Wing Kong, Gordon F. Tomaselli, Christopher S. Chen, Ronald A. Li

**Affiliations:** 1 Stem Cell & Regenerative Medicine Consortium, LKS Faculty of Medicine, The University of Hong Kong, Hong Kong; 2 Department of Physiology, The University of Hong Kong, Hong Kong; 3 Department of Bioengineering, Boston University, Boston, MA, USA; 4 Harvard Wyss Institute for Biologically Inspired Engineering, Boston, MA, USA; 5 Division of Cardiology, Department of Medicine, Johns Hopkins University, Baltimore, M.D., United States of America; 6 Dr. Li Dak-Sum Research Centre, The University of Hong Kong - Karolinska Institutet Collaborations in Regenerative Medicine, The University of Hong Kong, Hong Kong; 7 Ming Wai Lau Centre for Reparative Medicine, Karolinska Institutet, Sweden

## Abstract

Human embryonic stem cells (hESCs) is a potential unlimited *ex vivo* source of ventricular (**V**) cardiomyocytes (CMs), but hESC-**V**CMs and their engineered tissues display immature traits. In adult **V**CMs, sarcolemmal (sarc) and mitochondrial (mito) ATP-sensitive potassium (K_ATP_) channels play crucial roles in excitability and cardioprotection. In this study, we aim to investigate the biological roles and use of sarcK_ATP_ and mitoK_ATP_ in hESC-**V**CM. We showed that SarcI_K, ATP_ in single hESC-**V**CMs was dormant under baseline conditions, but became markedly activated by cyanide (CN) or the known opener P1075 with a current density that was ~8-fold smaller than adult; These effects were reversible upon washout or the addition of GLI or HMR1098. Interestingly, sarcI_K, ATP_ displayed a ~3-fold increase after treatment with hypoxia (5% O_2_). MitoI_K, ATP_ was absent in hESC-**V**CMs. However, the thyroid hormone T3 up-regulated mitoI_K, ATP,_ conferring diazoxide protective effect on T3-treated hESC-**V**CMs. When assessed using a multi-cellular engineered 3D ventricular cardiac micro-tissue (hvCMT) system, T3 substantially enhanced the developed tension by 3-folds. Diazoxide also attenuated the decrease in contractility induced by simulated ischemia (1% O_2_). We conclude that hypoxia and T3 enhance the functionality of hESC-**V**CMs and their engineered tissues by selectively acting on sarc and mitoI_K, ATP_.

Cardiovascular disease is a leading cause of death worldwide^1^. Myocardial infarction typically injures a large enough number of cardiomyocytes (CMs), subsequently leading to heart failure whose last resort is organ transplantation^2^. Human pluripotent stem cells, including embryonic stem cells (hESCs) and induced pluripotent stem cells (iPSC) are capable of self-renewal while maintaining their pluripotency to differentiate into all cell types including CMs. As such, hESC/iPSC provide a potentially unlimited *ex vivo* source of CMs for disease modelling, drug discovery, cardiotoxicity screening as well as cell replacement therapies. Despite such promises, it is now well accepted that hESC/iPSC-CMs and their engineered constructs are structurally and functionally immature with traits from weak contractile forces to higher susceptibility to apoptosis^3^,^4^,^5^,^6^,^7^,^8^,^9^,^10^,^11^ As such, there is a strong need to better understand the underlying basis of, and to derive effective strategies for driving maturation.

ATP-sensitive potassium (K_ATP_) channels, located on the sarcolemma and mitochondrial inner membranes of mature CMs, play an important role in cardioprotection during conditions such as ischemia by linking membrane excitability to metabolism^12^,^13^,^14^,^15^,^16^,^17^,^18^. Under physiological conditions, K_ATP_ channels are closed due to endogenous ATP inhibition^19^. When cytoplasmic ATP is depleted (e.g., during ischemia)^20^,^21^, sarcK_ATP_ opens to allow a repolarizing outward K^+^current, I_K, ATP_, thereby shortening the action potential (AP) and subsequently lowering Ca^2+^ influx to create a negative inotropic effect for metabolic conservation^14^,^22^,^23^,^24^. MitoK_ATP_, originally demonstrated through single channel recordings of ATP-sensitive K^+^currents in giant liver mitoplasts^25^. Was recently found to be composed of mitochondrial renal outer medullary potassium channel (ROMK) channel^26^. MitoK_ATP_ modulates ischemic preconditioning^27^,^28^, ischemic postconditioning^29^,^30^, and cytoprotection. Despite the crucial physiological role of K_ATP_ channels and the promises of hESC-CMs, neither sarc nor mitoI_K, ATP_ in hESC-CMs has ever been studied. The present study aims to fill this gap, while seeking biological insights and developing effective means for driving maturation of hESC-CMs.

## Result

### Expression of sarcK_ATP_ channel subunit transcripts in hESC-VCMs

**Figure 1:**
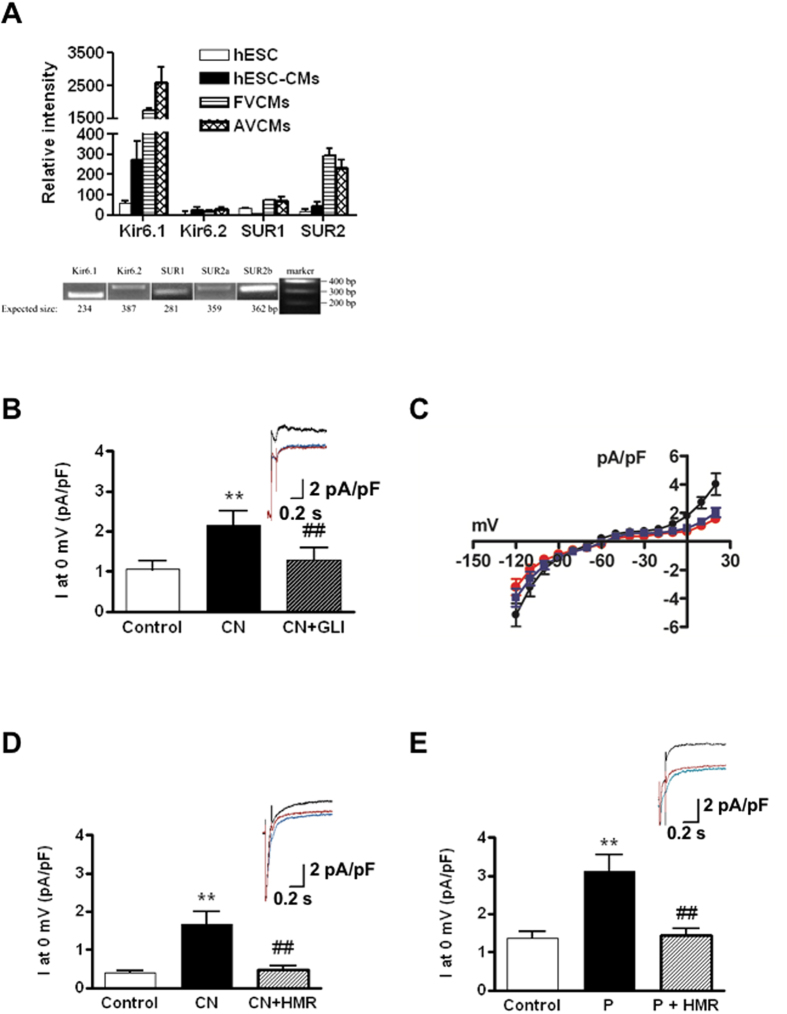
Expression of sarcolemmal K_ATP_ channels in hESC-VCMs. **(A)** *Top.* Transcriptomic profile of the key molecular components of sarcolemmal K_ATP_ channels in hESC, hESC-VCMs, fetal VCMs (F-VCMs) and adult VCMs (A-VCMs) as revealed by microarray. *Bottom.*RT-PCR validated the expression of sarcK_ATP_ channel subunits in hESC-VCMs. **(B**) *Top.* Representative current tracings of sarcolemmal I_K, ATP_ in hESC-VCMs at 0 mV under control conditions (blue line), with sodium cyanide (CN, 2 mM) alone (black line), and with CN and glibenclamide (GLI, 10 μM, red line). *Bottom*. Summary of averaged current densities under the same conditions. Cells were stimulated to 0 mV for 1000 ms from a holding potential of −80 mV preceded by a 100-ms prepulse to −10 mV. N = 8; **P < 0.01 compared to control group; ^##^*P* < 0.01 compared to CN group. **(C**) Current-voltage relationships of I_K, ATP_ under control conditions (blue line), with sodium cyanide (CN, 2 mM) alone (black line), and with CN and glibenclamide (GLI, 10 μM, red line). The membrane potential was normally held at −80 mV and the currents were evoked by a series of 1000 ms depolarizing and hyperpolarizing current steps (−120 mV to +20 mV in 10 mV steps). N = 7. **(D**) *Top.* Representative current tracings of sarcolemmal I_K, ATP_ in hESC-VCMs at 0 mV under control conditions (blue line), with sodium cyanide (CN, 2 mM, black line) alone, and with CN and HMR-1098 (HMR, 100 μM, red line). *Bottom.* Summary of averaged current densities under the same conditions. N = 9; **P < 0.01compared to control group; ^##^*P* < 0.01 compared to CN group. **(E)** *Top.* Representative current tracings of sarcolemmal I_K, ATP_ in hESC-VCMs at 0 mV under control conditions (blue line), with P-1075 (P, 100 μM, black line) alone, and with P and HMR-1098 (HMR, 100 μM, red line). *Bottom.* Summary of averaged current densities under the same conditions. N = 7; **P < 0.01compared to control group; ^##^*P* < 0.01 compared to P group.

As a first step, microarray-based transcriptomic analysis was performed to examine the expression of the 4 genes that encode for sarcK_ATP_ channels (KCNJ8 for K_ir_6.1; KCNJ11 for K_ir_6.2; ABCC8 for SUR1; and ABCC9 for the two splice variants SUR2A and SUR2B) in hESC-**V**CMs (Fig. 1A). All of the four genes were detected in hESC-**V**CMs, but the expression levels of K_ir_6.1, SUR1 and SUR2 were about 10-folds lower than those of hF- and hA-VCMs while K_ir_6.2 were comparably lowly expressed. RT-PCR confirmed that all sarcK_ATP_ channel subunits were detectable in hESC-**V**CMs (data not shown).

### Functional expression of sarcK_ATP_ channels in hESC-VCMs

Using these data as a guide, we functionally confirmed for the first time the existence of sarcI_K, ATP_ in hESC-**V**CMs. Electrophysiologically, sarcI_K, ATP_ was dormant at baseline (1.05 ± 0.22 pA/pF at 0 mV, n = 8) but could be markedly activated by the metabolic inhibitor cyanide (CN; 2 mM) which decreases ATP production via inhibition of cytochrome C oxidase in the electron transport chain of the mitochondria (current density = 2.15 ± 0.35 pA/pF at 0 mV, n = 8; p < 0.05) (Fig. 1B–C). CN-induced sarcI_K, ATP_ could be effectively and completely inhibited by the known sarcI_K, ATP_ blocker glibenclamide (10 μM) (1.27 ± 0.31 pA/pF at 0 mV, n = 8; Fig. 1B–C) or the specific inhibitor HMR1098 (100 μM) (0.47 ± 0.11pA/pF at 0 mV, n = 9; Fig. 1D). Similarly, sarcI_K, ATP_ in hESC-VCMs could be activated by the specific sarcI_K, ATP_ opener P1075 (100 μM) (3.11 ± 0.45 pA/pF at 0 mV, n = 7) and inhibited by HMR1098 (1.43 ± 0.19 pA/pF at 0 mV, n = 7) (Fig. 1E).

### Functional effect of sarcK_ATP_ channel mediators on the AP phenotype of hESC-VCMs

**Figure 2:**
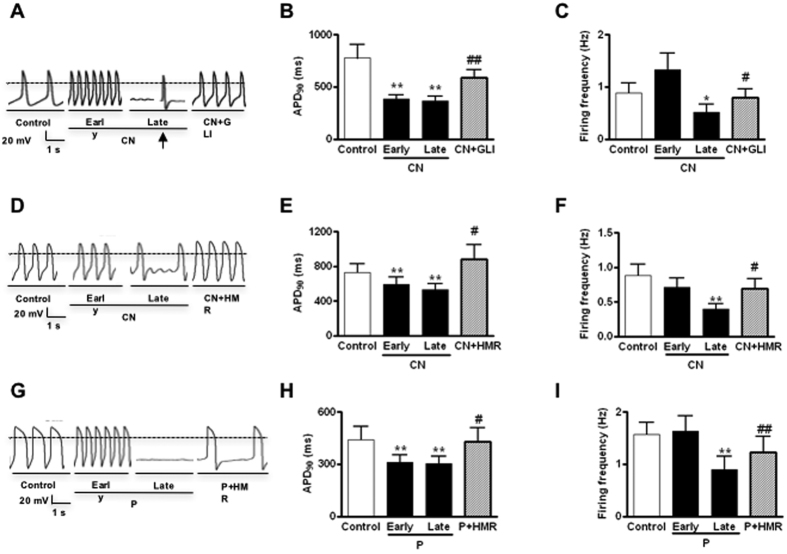
Effects of the sarcolemmal I_K, ATP_ blocker HMR1098 and opener P-1075 on the action potential properties of hESC-VCMs. **(A**) Representative action potential tracings of hESC-VCMs before and after sodium cyanide (CN, 2 mM) treatment with or without glibenclamide (GLI, 10 μM). Early CN defined as CN treatment at the beginning (2–3 min). Late CN defined as steady-state after CN treatment. For this particular cell (12 cells), AP firing ceased in late CN application. Others (13 cells) showed significantly reduced firing frequencies. The arrow indicates an electrical stimulation (200 pA, 5 ms). **(B,C**) Bar plots summarizing the effects on AP parameters (**B**) APD_90_, (**C**) Firing Frequency N = 25; *P < 0.05, **P < 0.01compared to control; ^#^*P* < 0.05, ^##^*P* < 0.01 compared to late CN. (**D)** Representative action potential tracings of hESC-VCMs before and after sodium cyanide (CN, 2 mM) treatment with or without 100 μM HMR-1098 (HMR). **(E,F**) Bar plots summarizing the effects on AP parameters. (**C**) APD_90_, (**D**) Firing Frequency. N = 18; *P < 0.05, **P < 0.01compared to control; ^#^*P* < 0.05, ^##^*P* < 0.01 compared to late CN. **(G**) Representative action potential tracings of hESC-VCMs before and after P-1075 (100 μM) treatment, with or without 100 μM HMR-1098. AP firing ceased 5–10 minutes after P application (Late) in 4 of 14 cells. Others (10 cells) showed significantly reduced firing frequencies. **(H,I**) Bar plots summarizing the effects on AP parameters. G) APD_90_, (**H**) Firing Frequency N = 14; *P < 0.05, **P < 0.01compared to control group; ^#^*P* < 0.05, ^##^*P* < 0.01 compared to late P-1075 group.

As previously published, spontaneous AP firing is a developmental hallmark of immature hESC-**V**CMs^5^. Upon the addition of CN (2 mM), APD at 90% repolarization (APD_90_) significantly shortened (from 775.6 ± 129.7 ms to 385.3 ± 40.5 ms, n = 25, *P* < 0.05) (Fig. 2A,B), thereby hastening the spontaneous AP firing (0.88 ± 0.19 vs 1.33 ± 0.32 Hz in control) (Fig. 2C). Other AP parameters such as the amplitude, upstroke velocity, decay velocity or maximal diastolic potential were not significantly altered (Table 1). Subsequently at steady-state, half of the CN-treated hESC-**V**CMs completely ceased firing (n = 12 of 25); the frequency of the remaining AP-firing cells reduced to 0.52 ± 0.16 Hz. For the CN-silenced cells, an AP could be elicited when given a single depolarizing stimulus, indicating the excitability of CN-treated cell remained intact (Fig. 2A). All CN effects were readily reversible upon washout or with the addition of glibenclamide (10 μM) (Fig. 2A–C) or HMR1098 (Fig. 2D–F). As anticipated from the defined action of P1075 on sarcI_K, ATP_, similar effects on AP firing and properties which could be reversed by HMR1098 were observed (Fig. 2G–I).

### Lack of effect of mitoK_ATP_ channel mediators on sarcI_K, ATP_ and AP phenotype

**Figure 3:**
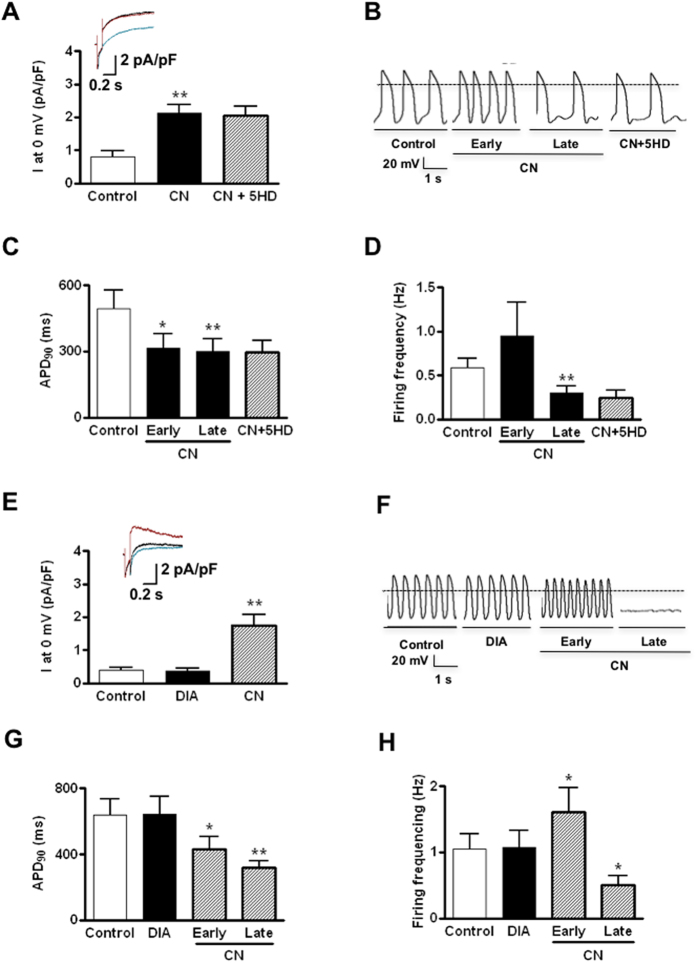
Effects of the mitochondrial I_K, ATP_ blocker 5-HD and opener Diazoxide on the action potential properties of hESC-VCMs. **(A**) *Top.* Representative current tracings of sarcolemmal I_K, ATP_ in hESC-VCMs at 0 mV under control conditions (blue line), with sodium cyanide (CN, 2 mM, black line) alone, and with CN and 5-hydroxydecanoate (5HD, 100 μM, red line). *Bottom.* Summary of averaged current densities under the same conditions. N = 6; **P < 0.01compared to control group. **(B**) Representative action potential tracings of hESC-VCMs before and after sodium cyanide (CN, 2 mM) treatment with or without 100 μM 5-hydroxydecanoate (5HD). **C,D**) Bar plots summarizing the effects on AP parameters. N = 13; *P < 0.05, **P < 0.01compared to control group. (**E**) *Top.* Representative current tracings of sarcolemmal I_K, ATP_ in hESC-VCMs at 0 mV under control conditions (blue line), with diazoxide (DIA, 100 μM, black line), or sodium cyanide (CN, red line). *Bottom.* Summary of averaged current densities under the same conditions. N = 7; **P < 0.01 compared to control group. **(F**) Representative action potential tracings of hESC-VCMs before and after diazoxide (100 μM) treatment, followed by sodium cyanide (2 mM) application. AP firing ceased 5–10 minutes after CN application (Late) in 9 of 18 cells. Others (9 cells) showed significantly reduced firing frequencies. **(G,H**) Bar plots summarizing the effects on AP parameters. N = 18; **P < 0.01 compared to control group.

In stark contrast to CN, GLI, P1075 and HMR1098, CN-mediated changes in sarcI_K, ATP_ and AP were not altered by the mitoI_K, ATP_ blocker 5HD (100 μM) (Fig. 3A–D). Unlike CN, the mitoI_K, ATP_ opener diazoxide (100 μM) could not activate sarcI_K, ATP_ (Fig. 3E) and had no effect on AP firing and parameters (Fig. 3F–G and Table 1).

### Hypoxia drives sarcI_K, ATP_ maturation in hESC-VCMs

**Figure 4:**
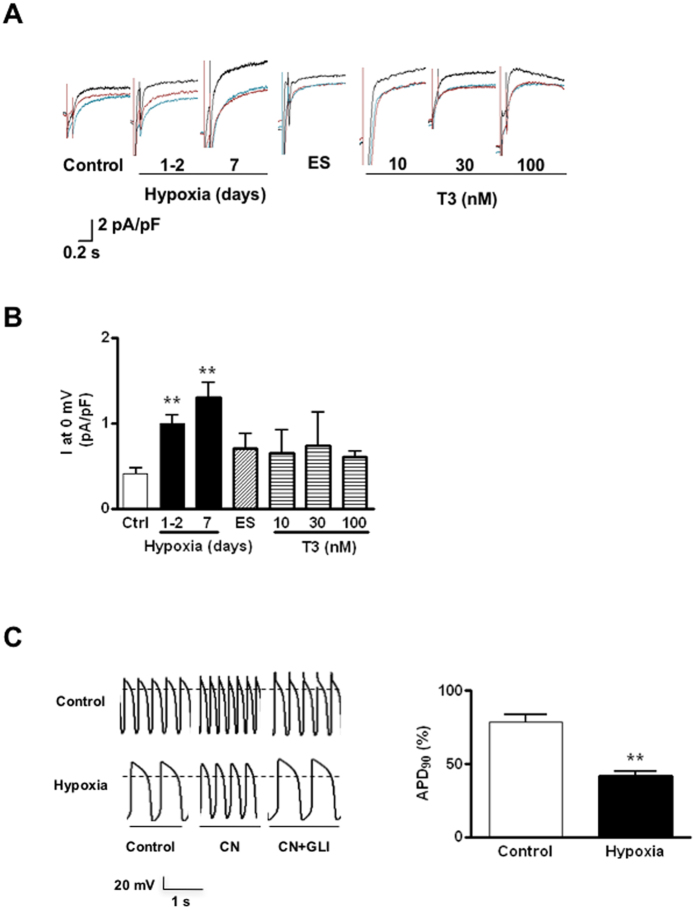
Driven maturation of I_K, ATP_ in hESC-VCMs and the resultant functional consequences. **(A**) Representative sarcolemmal I_K, ATP_ tracings, recorded in the absence (blue line) or presence of cyanide (CN, 2 mM, black line) with or without glibenclamide (GLI, 10 uM, red line) at 0 mV, of hESC-VCMs treated with hypoxia (5% O_2_ for 24–48 hrs, 7 and 14 days), electrical field stimulation (ES, 1 Hz for 14 days) or T3 (10, 30, 100nM for 2 days). **(B**) Bar plots summarizing the I_K, ATP_ densities at 0 mV under the corresponding conditions. **P < 0.01. N = 6, 12, 8, 5, 4, 2, 3, 2 for controls, hypoxia for 24–48 hrs, 7 days, ES, or 10, 30 or 100 nM T3 for 2 days. **P < 0.01 compared to controls. **(C**) Representative action potential tracings and bar plots summarizing the effects on AP parameters of hESC-VCMs subjected to hypoxia (5% O_2_) for 7 days. The percentage of APD shown in the bar plots is the ratio of APD in CN treatment over APD in CN with GLI treatment. N = 4 and 7 for controls and hypoxia for 7 days. *P < 0.05, **P < 0.01.

Given the well-defined physiological role of sarcI_K, ATP_ as a metabolic sensor in adult CMs^20^,^21^,^22^,^23^,^24^, we hypothesized that sarcI_K, ATP_ in hESC-**V**CMs would respond to oxygen tension and change its expression accordingly^21^,^31^. To test this postulation, we subjected hESC-**V**CMs to a hypoxic condition (5% vs.20% O_2_) then compared their sarcI_K, ATP_ and AP changes in response to sarcI_K, ATP_ mediators to time-matched (normoxic) controls. Figure 4A,B shows that hypoxia time-dependently upregulated sarcI_K, ATP_ with a ~3-fold increase in its current density at day 7. In contrast, neither electrical conditioning (at 1 Hz for 14 days) that we previously showed to mature AP phenotype (by up-regulating I_K1_)^5^ nor thyroid hormone treatment (10–100 nM triiodothyronine, T3, for 2 days) significantly altered sarcI_K, ATP_. As for the AP phenotype, CN significantly shortened APD_90_ and hastened AP firing of hypoxia-treated hESC-VCMs compared to un-treated controls, consistent with an up-regulated sarcI_K, ATP_ (Fig. 4C). As anticipated, CN effect could be reversed by GLI.

### MitoK_ATP_ was absent in untreated hESC-VCMs

**Figure 5:**
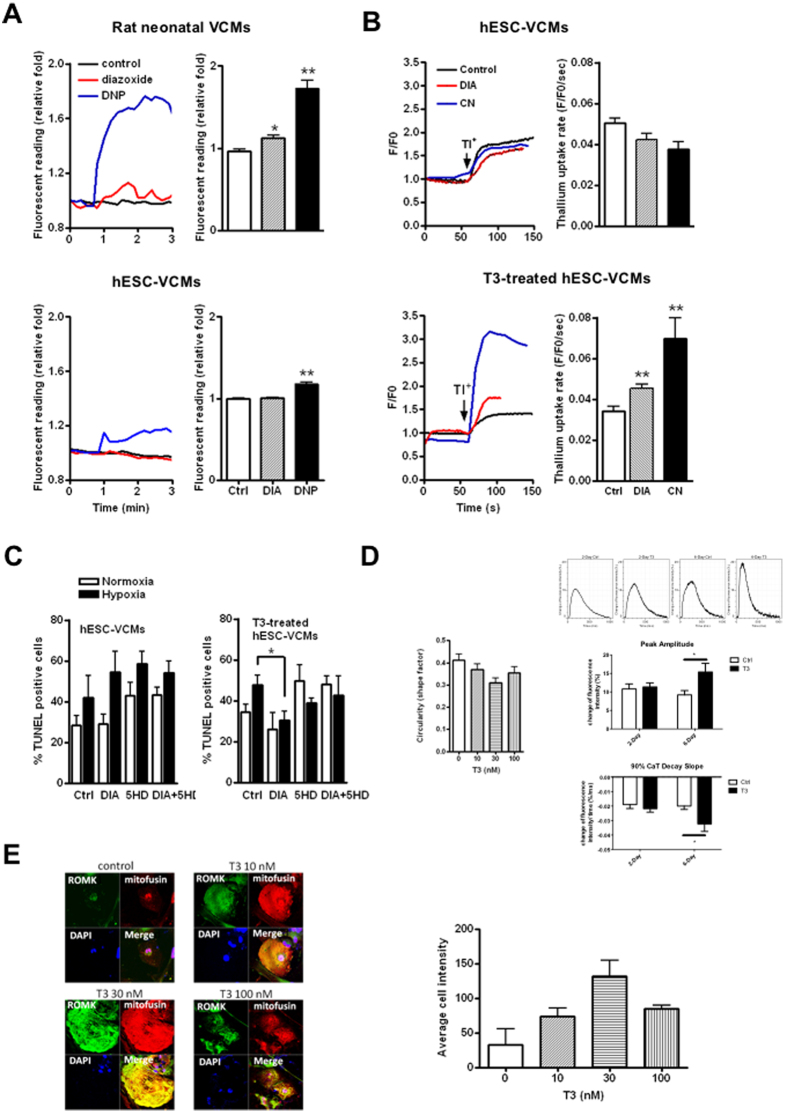
Role and driven maturation of mitochondrial I_K, ATP_ activity in hESC-VCMs. **(A**) Time course of changes in flavoprotein fluorescence in neonatal rat VCMs and hESC-VCMs exposed to DNP (100 μM, blue), diazoxide (DIA, 100 μM, red) and control (black) buffer as indicated. N = 4 for each. (**B)** Thallium assays in hESC-VCMs with or without T3 treatment (100 nM for 2 days). The representative tracing and summarized thallium uptake rate of hESC-VCMs or T3-treated hESC-VCMs exposed to control (black) buffer, diazoxide (DIA, 100 μM, red) or cyanide (CN, 2 mM, blue). N = 79, 40 and 29 for control, DIA and CN in hESC-VCMs, 26, 68 and 25 for control, DIA and CN in T3-treated hESC-VCMs. **P < 0.01. (**C)** TUNEL assay of normoxia and hypoxia (1% O_2_)-treated hESC-VCMs and 300 nM T3-treated hESC-VCMs, in the presence of diazoxide (100 μM) with or without 5HD (1 mM) as indicated. N = 8. *P < 0.05. **(D**) Cell circularity and APD_90_ in hESC-VCMs after T3 treatment for 2 and 6 days. N = 16 and 26 for 2 days and 6 days respectively. *P < 0.05. **(E)** ROMK expression in hESC-VCMs after T3 treatment. Mitofusin-2 is shown as a mitochondrial marker.

MitoK_ATP_ signal was detected by confocal imaging of flavoprotein fluorescence. In the positive control isolated neonatal rat VCMs, both the non-specific opener DNP and the mitoK_ATP_ specific opener diazoxide significantly increased flavoprotein fluorescence reading by about 1.7- and 1.1-fold, respectively when compared to controls (Fig. 5A), consistent with previously reported data^28^. By contrast, DNP increased the fluorescence of hESC-VCMs by only ~1.2 fold relative to controls. Diazoxide did not result in any significant changes (Fig. 5A), indicating that mitoK_ATP_ channels were not functionally present in hESC-VCMs. These results were further confirmed by thallium uptake assay^26^. Neither diazoxide nor CN increased the thallium uptake rate of hESC-VCMs (Fig. 5B).

We next investigated the cardioprotective effect of mitoK_ATP_ channels during simulated ischemic (1% O_2_) by TUNEL assay. Neither DNP nor diazoxide could protect control hESC-VCMs from simulated ischemia-induced cell death. 5HD also did not lead to further cell death compared to their non-treated counterparts, suggesting that mitoK_ATP_ channel did not open to confer cardioprotection during ischemic/hypoxic insult (Fig. 5C and Supplemental Figure 1), consistent with the observation that functional mitoK_ATP_ is absent in hESC-VCMs.

### Driven maturation of mitoK_ATP_ channels by triiodothyronine in hESC-VCMs

Since the thyroid hormone T3 is known to be involved in regulation of expression of cardiac genes^32^ and sensitivity of K_ATP_ channels to ATP^33^, we attempted to investigate the effect of T3 treatment on mitoK_ATP_ related actions in hESC-**V**CMs. When treated with T3 (100 nM) for 48 hrs, the cell circularity, a surrogate index of **V**CM maturity, of hESC-**V**CMs decreased (Fig. 5D). T3-treated hESC-**V**CMs also showed a progressive increase in calcium transient peak amplitude and 90% decay velocity from day 2 to day 6 post treatment, consistent with a more mature calcium transient profile (Fig. 5D). This is further corroborated by an increase in expression of key calcium handling proteins including SERCA2a and phospholamban (data not shown). RNAseq analysis of T3-treated hESC-**V**CMs showed an increase in the expression of SUR2. which has been shown to be a subunit of mitoK_ATP_^34^, while other subunits including Kir 6.1, Kir 6.2 and SUR1 was found to have decreased in expression (data not shown). Interestingly, T3 treatment also increased the expression of ROMK (Fig. 5E), the major molecular constituent of mitoK_ATP_^26^. Both diazoxide and CN significantly increased the thallium uptake rate in these T3-treated hESC-**V**CMs (Fig. 5B), demonstrating an increase in mito I_K, ATP_. Moreover, when treated with T3 for 48 hrs followed by a simulated ischemic insult for 8 hours, the % of TUNEL positive cells decreased in the presence of diazoxide (Fig. 5C). This effect could be inhibited by 5-HD. In contrast, the specific sarcK_ATP_ opener P1075 did not decrease the % of TUNEL positive cells after simulated ischemic insult. The specific sarcK_ATP_ inhibitor HMR1098 also showed no effect (Supplemental Figure 3). Interestingly, hypoxia and electrical field stimulation did not enhance mitoK_ATP_ channels in hESC-VCMs, indicating a cue-specific effect.

### Effects of T3 on engineered human ventricular cardiac microtissues (hvCMT)

**Figure 6:**
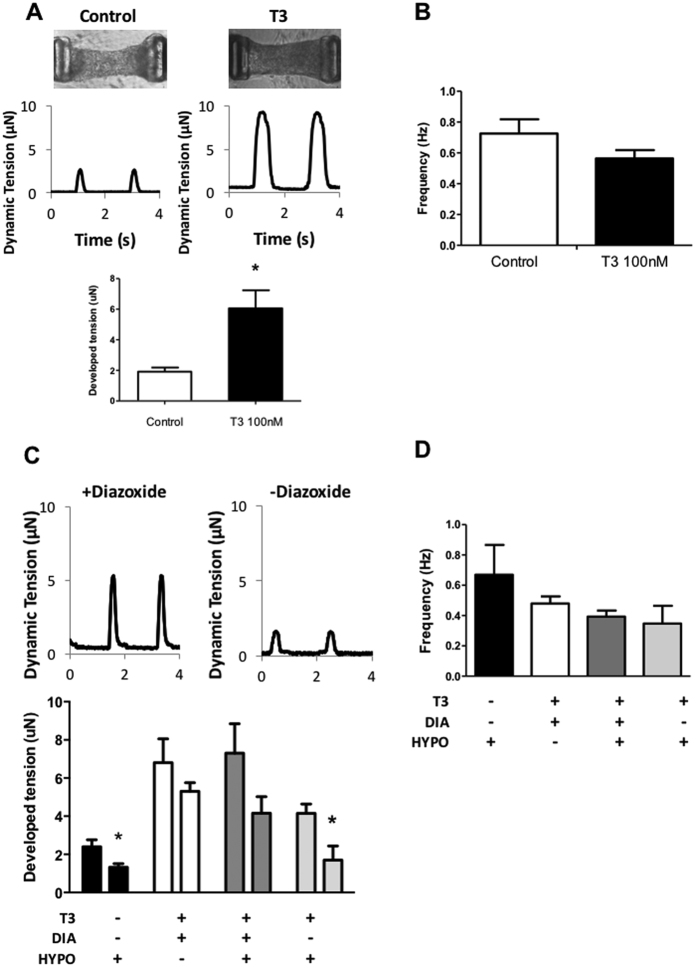
Role and driven maturation of mitochondrial I_K, ATP_ activity in hvCMTs. **(A)** Developed tension of hvCMTs with or without T3 treatment for 6 days. Top. Representative tracing of tension changes during spontaneous contraction of hvCMTs. Bottom. Bar graph showing average developed tension in control and T3 treated hvCMTs during spontaneous contraction. N = 6 for both control and T3. p < 0.05. (**B**) Spontaneous contraction frequency in control and T3 treated hvCMTs at day 6 after seeding. N = 6 for both control and T3.(**C**) Developed tension of hvCMTs with or without T3 treatment after simulated ischemic insult. Top. Representative tracing of tension changes during spontaneous contraction of hvCMTs after 8 hrs hypoxia (1%O_2_) and reoxygenation treatment. Bottom. Bar graph showing average developed tension in hvCMTs before and after hypoxia/reoxygenation treatment. N = 4 for all groups. p < 0.05. (**D**) Spontaneous contraction frequency in control and T3 treated hvCMTs at day 6 after seeding. N = 4 for all groups.

To exploit the potential use of T3 for driven tissue maturation, we next examined the functional consequences of their treatments on a multi-cellular 3D ventricular cardiac microtissue (hvCMT) system, where true dynamic tension developed by the tissue in real time, rather than shortening of single hESC-CMs or their clusters as an surrogate index for contractile forces^35^,^36^, can be measured. We reasoned that the improved calcium transient and cell survival induced by T3 would translate into stronger contractile forces at the tissue level, particularly after hypoxic insult. Figure 6A shows that hvCMT engineered from approximately 1000 hES2-**V**CMs each of ~0.3 mm in length which allowed continuous measurement of their dynamic twitch tension. To allow for thorough compaction of the tissues, hvCMTs were allowed to culture for 6 days with or without T3 treatment before dynamic twitch tensions were measured. The developed twitch tension in T3 –treated, time-matched hvCMTs increased significantly (by 3-fold, P < 0.01, n = 6). However, the spontaneous twitch frequency was not altered by T3 (P > 0.05) (Fig. 6B). Consistent with an improved cell survival of T3-treated cells, T3-treated hvCMT displayed an attenuated decrease in developed tension after being subjected to simulated ischemia insult (1%O_2_) when treated with the mitoK_ATP_ opener diazoxide (Fig. 6C). The spontaneous twitch frequency was unaffected by T3 and diazoxide treatment (Fig. 6D).

## Discussion

The roles of sarc and mito I_K, ATP_ in excitability, cell viability and cardioprotection of adult CMs are well established, but have not been tested in hESC-VCMs and their engineered tissues. In the present study, we tested the hypothesis that I_K, ATP_ endows upon hESC-VCMs and their engineered tissues resilience to metabolic demand and ischemic insult. We found that although sarcK_ATP_ was expressed at a relatively low basal level in hESC-**V**CMs (~1/8 of adult), upon partial activation it was sufficient to cause APD shortening and therefore accelerated AP firing; when fully activated, sarcK_ATP_ of hESC-**V**CMs even silenced automaticity without compromising the intrinsic cellular excitability. These effects were even more prominent after hypoxia-driven augmentation of sarcK_ATP_ (by ~3-fold). By contrast, neither T3 treatment nor electrical conditioning (data not shown) affected sarcI_K, ATP_; this mirrored the upregulation of mito I_K, ATP_ by T3 but not hypoxia, indicating cue-specific effects on sarc and mito I_K, ATP_. For hypoxia-treated hESC-**V**CMs, AP parameters were not changed under baseline condition when sarcoI_K, ATP_ was dormant^19^. Sarcolemmal KATP has long been shown to be involved in cardioprotection during metabolic stress including ischemia/reperfusion^21^. While more recent studies point to mK_ATP_ as the key player in cardioprotection in ischemic^37^, sarcK_ATP_ has been shown to have an increased expression during preconditioning, with the increase expression of SUR2A. Thus, though the role of sarcolemmal K_ATP_ in cardioprotection is controversial, our results which demonstrates that sarcolemmal K_ATP_ expression is increased in hESC-CMs after 2 days of hypoxia is in accord with previous studies in isolated cardiomyocytes. It has been shown that all subunits of K_ATP_ channels are expressed in low levels during fetal and prenatal phase, and continue to increase in expression after birth^38^. Chronic mild hypoxia has also been shown to increase SarcK_ATP_^31^. Our observation that SarcK_ATP_ is increased in hypoxia may not serve as a pre-conditioning signal but rather in response to a developmental cue.

Unlike sarcK_ATP,_ mitoI_K, ATP_ was not functionally expressed in hESC-VCMs. The protein of its molecular correlate ROMK was also absent. Given the defined role of mitoI_K.ATP_^25^,^26^, these findings may provide a potential explanation, at least in part, for the poor graft survival after CM transplantation^39^,^40^ since the recipient milieu is often hostile and ischemic. Interestingly, upon T3 treatment, but not hypoxia, the expression of ROMK protein and mitoK_ATP_ were induced, along with an improved survival of hESC-**V**CMs after hypoxic insult. T3 treated engineered ventricular tissues also maintained their contractile force after ischemic/hypoxic insult in the presence of the specific mitoK_ATP_ opener diazoxide, confirming that the cardioprotective effect is conferred by mitoK_ATP_. The contractile forces of engineered ventricular tissue constructs were also enhanced after T3 treatment although the automaticity was not affected. Previous studies reported that T3 shortened action potential duration by decreasing L-type calcium channel (I_ca-L_)^41^,^42^ and increasing transient outward potassium (I_to_)^43^ and ultrarapid potassium (I_Kur_)^42^ current density. However, such was not observed in hESC-VCMs even one week after T3 (100 nM) treatment (Supplemental Figure 2). Indeed, RNA-seq data on hESC-VCMs reveals that the expression of L-type calcium channels was increased while the expression of Kir 2.1 was found to be unchanged after 7 -days T3 treatment (data not shown). The increase in contractile forces observed in engineered tissues after T3 treatment is likely due to the demonstrated improvement in calcium handling, which is a well established role of thyroid hormone and is independent of the cardioprotective effects conferred by mitoK_ATP_. However, both effects are likely to be genomic in nature as long term treatment with T3 (in a matter of days) is required for these effects to be observed and that the effects persist after the removal of T3. The level of the thyroid hormone receptors TRα and TRβ are also observed to increase with T3 treatment (data not shown).

Collectively, our data suggest that sarcK_ATP_ is a prime candidate to target for electrophysiological stability and maturation, while mito I_K, ATP_ engineering would improve the longevity of engraftment. We conclude that hypoxia and T3 enhance the functionality of hESC-**V**CMs and their engineered tissues by selectively acting on sarc and mitoI_K, ATP_. The results further implicate that multiple micro-environmental signaling cues uniquely interact to alter the expression and function of different gene products of hESC-VCMs. Therefore, a combinatorial approach will likely be needed for deriving more effective methods to drive maturation of hESC/iPSC-VCMs and their engineered tissues to a more adult-like state. A better understanding of the underlying key players and mechanisms is crucial to accomplish this goal.

## Methods

### Human ESC culturing and directed cardiac differentiation

Undifferentiated hESC (hES2) were maintained in mTeSR™1 on matrigel (BD)-coated plates. Direct cardiac differentiation was initiated with a modified embryoid body formation protocol^44^. To initiate cardiac differentiation, hESCs were dissociated into single cells using Accutase (Invitrogen) and cultured in mTeSR^TM^1 medium with Matrigel^TM^ (40 μg/ml), BMP-4 (1 ng/ml, Invitrogen) and Rho kinase (ROCK) inhibitor (10 μM; R&D) under hypoxic (5% O_2_) condition. Twenty-four hours later, the culture was washed and replaced in StemPro34 SFM (Invitrogen) with ascorbic acid (AA, 50 μg/ml; Sigma), 2 mM GlutaMAX-1 (Invitrogen), BMP4 (10 ng/ml) and human recombinant activin-A (10 ng/ml; Invitrogen) for 3 days. On day 4, IWR-1, a small molecule Wnt inhibitor, (5 μm/ml; Enzo Life Sciences) was added to inhibit the canonical Wnt signaling. On day 8, cells were transferred to normoxic environment and maintained in StemPro34 SFM + AA medium. Around 20 days post cardiac differentiation, cardiac clusters were dissociated for 5 min by 0.05% trypsin in PBS without calcium and magnesium, followed by transferring to Dulbecco’s Modified Eagle Medium (DMEM, Invitrogen) with 5% fetal calf serum, 2 mML-glutamine, 1% penicillin/streptomycin and 100 μM non-essential amino acids solution (Invitrogen). The cultures were maintained under an atmosphere of water saturated 5% CO_2_/95% air at 37 °C. Twenty-four hours later, cells were transduced with the lentiviral construct LV-MLC2v-tdTomato-T2A-Zeo that we previously published and described^7^. Three days after transduction, zeocin (300 μg/ml) was used to select for hESC-**V**CMs. The selected VCMs were used for downstream experiments at 30 days post-differentiation.

### Isolation of human fetal and adult ventricular CMs

Human fetal and adult left ventricular CMs were isolated in accordance to regulations, guidelines and protocols approved by the UC Davis IUPAC and IRB Protocol (#200614787-1 and #200614594-1) with written informed consent given for the use of the human tissue.

All fetal (18–20 weeks) and adult (50–70 years) hearts were digested using the Langendorff system with a recirculatory system that circulated the 37 °C collagenase solution until cells started to dissociate into the enzyme solution. The fetal hearts typically took about 30min and the adult hearts took over 3 hours to digest. The collagenase solution contained 200 U/ml collagenase II (Worthington Biochemical Corp), 4 mg protease (Sigma) with 1% BSA. After enzyme treatment, the hearts were chopped manually to release the cells into high K^+^ solution. The fetal cells were plated for 1 hour in M199 with 5 mM carnitine, 5 mM creatine, 5 mM taurine, 10% FBS and 1% penicillin/streptomycin to remove fibroblasts. The medium was then collected to retrieve the CMs in suspension. The adult cells were not plated but allowed to settle by gravity for 15 min. The denser CMs at the bottom of the conical tubes were collected.

### Isolation of rat neonatal ventricular cardiomyocytes

The use of animals and all experimental protocols in this study were approved by the Committee on the Use of Live Animals in Teaching and Research of the University of Hong Kong. All animal experiments were performed in accordance with approved guidelines. Hearts from 2-day-old neonatal rats were isolated and rinsed in ice-cold phosphate-buffered saline (PBS) solution. After removal of the atria, the ventricles were minced with scissors and washed with ice-cold PBS washed three times in ice-cold PBS and digested with PBS containing DNase (0.025% w/v), collagenase (0.1%), and trypsin (0.05%) on a rotary shaker at 37 °C for 60 min. The digested cells were pelleted by centrifugation at 300 g and was resuspended in DMEM medium containing 5% fetal bovine serum, 10% horse serum, 50 μg/ml gentamicin) for differential plating. After serial plating, the resulting pellet was resuspended in plating media. The cells were plated at a density of 1.8–2.0 × 10^6^ cells/plate.

### Transcriptomic profiling

Microarray experiments were performed in hESC, hESC-**V**CMs, human fetal VCMs (hFVCMs) and human adult VCMs (hAVCMs). Cell samples were suspended and lysed in Trizol (Invitrogen). After adding 1:4 volume chloroform, aqueous and organic phases were separated using heavy PLG tubes (Eppendorf). Sentrix WG-6 beadchips (Illumina, San Diego, CA) were used to profile mRNA expression. Microarray data were analyzed using the BeadStudio for transcriptomic (Illumina) software packages. Expression was normalized using background subtraction and cubic spline (BeadStudio) or composite LOESS normalization (WebArrayDB).

### Reverse transcription polymerase chain reaction (RT-PCR)

RNA of hESC-**V**CMs was extracted by RNeasyPlus Mini Kit and cDNA was synthesized by QuantiTect Reverse Transcription Kit according to product protocol. AccuPrime^TM^PfxSuperMix (Invitrogen) was used to perform PCR reactions by using the following primers: Kir6.1 (forward: CTGGCTGCTCTTCGCTATC and reverse: AGAATCAAAACCGTGATGGC), Kir6.2 (forward: TGTCCCGCAAGGGCATCATCCCCG and reverse: TAGTCACTTGGACCTCAATGGAG), SUR1 (forward: CGATGCCATCATCACAGAAG and reverse: CTGAGCAGCTTCTCTGGCTT), SUR2A (forward: ATATGGTCAAATCTCTACCTGGAGG and reverse: GTTGGTCATCACCAAAGTGGAAAAG) and SUR2B (forward: ATATGGTCAAATCTCTACCTGGAGG and reverse: CATGTCTGCGCGAACAAAAGAAGC).

### Electrophysiology

The K_ATP_ channel currents and action potentials were recorded by whole-cell patch-clamp studies in hESC-**V**CMs at 37 °C using the EPC10 amplifier and Pulse/PulseFit software (HEKA, Germany) as previously described^12^. Patch electrodes (3.0–5.0 MΩ) were filled with the internal pipette solution containing 120 mM potassium glutamate, 25 mMKCl, 1 mM ATP (magnesium salt), 10 mM EGTA, 0.5 mM MgCl_2_ and 10 mM HEPES (pH 7.2). The external bath solution contained 140 mMNaCl, 5 mMKCl, 1 mM MgCl_2_, 1 mM CaCl_2_ and 10 mM HEPES (pH 7.4). Glibenclamide (Sigma-Aldrich), diazoxide (Sigma-Aldrich), 2, 4-dinitrophenol (Sigma-Aldrich) and P1075 (Santa Cruz Biotechnology) were dissolved in DMSO before they were added to experimental solutions. Sodium cyanide (Sigma-Aldrich), 5-hydroxydecanoic acid (Sigma-Aldrich) and HMR 1098 (Axon Medchem BV) were dissolved directly in external bath solution.

### Confocal Imaging of flavoprotein fluorescence

The auto-fluorescence of mitochondrial flavoprotein protein was measured by Zeiss LSM700 confocal microscope with a 63x oil objective. Excitation wavelength of the solid state laser was set at 488 nm and emission was collected after 515 nm. Images were taken every 10 seconds.

### Thallium Uptake Assay

Prior to the assay, cells were loaded with a fluorescent indicator, Fluozin-2 AM (5 μM) (Invitrogen). The dye is sensitive to thallium, which is used to substitute for potassium. Dye was loaded in its acetoxymethyl ester (AM) form mixed with 0.2% (w/v) Pluronic F-127 prior to addition to the incubating medium. Cells were incubated with Fluozin-2 AM at 37 °C for 20 min, followed by two PBS washes to remove membrane bound dye. Just prior to the TI^+^ assay, cells were treated with 150 μM digitonin for 30 seconds to permeabilize the plasma membrane, as evidenced by rapid loss of the cytosolic component of dye, leaving only the mitochondrial compartment loaded with the indicator.

Images were collected by exciting at 488 nm and fluorescence was recorded by using Zeiss LSM700 confocal microscope. Bath TI^+^ was rapidly switched from 0 to 2 mM TI^+^ at 70 seconds. The chloride-free, 0 mM TI^+^ assay bath solution contained (in mM): 195 mannitol, 10 HEPES, 2 MgSO_4_, 2 Na_2_HPO_4_, 2 succinate, 0.6 CaOH and1 EGTA (pH 7.2) and the 2 mM TI^+^ solution was identical except 2 mM TISO_4_ was added. Images were collected every 1.56 seconds, and the fluorescence intensities of the dyes were determined offline by ImageJ (NIH, USA). F/F0 was calculated for each timepoint (F0 was the basal fluorescence in the 0 Tl^+^ condition).

### TUNEL assay on hypoxia

The hESC-VCMs or rat neonatal ventricular cardiomyocytes were seeded onto 96-well plates for 24 hours to allow cell attachment. Cells were given T3 (100 nM) treatment for 2 days after which either vehicle or diazoxide (1 uM) were added before they were transferred to a hypoxic chamber maintained at a 1% O_2_/5% CO_2_/95% N_2_ environment for 8 hours to simulate ischemia. The percentage of cell death was detected by TUNEL assay with the *In Situ* Cell Death Detection kit (Roche Biosciences, U.S.A). Briefly, the plates were washed with PBS and fixed in paraformaldehyde solution (4% in PBS) for 15 min at room temperature. The cells were permeabilized in a solution containing 0.1% Triton X-100 in 0.1% sodium citrate for 2 min on ice, followed by incubation in freshly prepared TUNEL reaction mixture for 60 min at 37 °C in the dark. The plates were washed with PBS and counterstained with the DAPI for identification of nuclei. Samples were then imaged with Nikon Eclipse TiS microscope (Nikon Instrument Inc, Melville, NY, USA) and analyzed with Image J (NIH, USA).

### Immunostaining

For staining of hESC-VCMs. adherent cells were fixed for 15 min at room temperature with 4% paraformaldehyde in phosphate-buffered saline (PBS). After washing with PBS, cells were permeabilized in PBS containing 1% Triton X-100 and subsequently blocked in 3% bovine serum albumin. Mitofusin-2 and ROMK1 (KCNJ1) antibodies were purchased from Abcam (Cambridge, MA, USA). Primary antibodies were diluted in PBS with 1% BSA at 1:200 and incubated at RT for 2 hours. Alexa Fluor (AF) 488-conjugated goat anti-rabbit IgG or AF555 anti-mouse IgG (Invitrogen) were used as secondary antibodies and stained for 1 hour at RT. Coverslips were mounted onto glass slides in Prolong Gold mounting medium with DAPI (Invitrogen) and samples were imaged on LSM Carl Zeiss 510 Meta (Carl Zeiss, Jena, Germany). Images were then analyzed and circularity index for each cell given by Image J (NIH, USA).

### Quantification of contractile force in human ventricular cardiac microtissues (hvCMT)

Human ventricular cardiac microtissues were prepared as previously described^45^. In brief, Polydimethylsiloxane (PDMS, Sylgard 184, Dow-Corning) microfabricated tissue gauges (μTUG) substrates were molded from the SU-8 masters, with embedded fluorescent microbeads (Fluoresbrite 17147; Polysciences, Inc.) on the cantilever ends. Human ESC-VCMs were dissociated after trypsin digestion at 14–20 days post differentiation. The unpurified dissociated cells were used to produce microtissues and is composed of >80% cTnT as determined by FACS analysis. A suspension of ~10^6^ cells within the reconstitution mixture, consisting of 1.5 mg/mL liquid neutralized collagen I (BD Biosciences) and 0.5 mg/mL fibrinogen (Sigma-Aldrich), was added to the substrate and the entire assembly was centrifuged to drive the cells into the micropatterned wells, where hESC-VCMs self-assembled into microtissues within 24 hr. For quantifying microtissue forces, brightfield and fluorescence images were taken at 100 Hz with a fast CCD camera (Allied Vision), and an A-Plan 10x objective on a Nikon Eclipse Ti (Nikon Instruments, Inc.) equipped with a live cell incubator. Only tissues that were uniformly anchored to the tips of the cantilevers were included in the analysis. The displacement of fluorescent microbeads at the top of the cantilevers was then tracked with using the SpotTracker plug-in in ImageJ (National Institutes of Health). Microtissues were treated with the thyroid hormone triiodothyronine (T3, 100 nM) at the time of seeding for 6 consecutive days. Measurements were made on day 6 of T3 treatment. Microtissues were then transferred to a hypoxic chamber maintained at a 1% O_2_/5% CO_2_/95% N_2_ environment for 8 hours to simulate ischemia. They were then placed back to a normoxic environment for 8 hours after which force measurements on the same tissues were made again.

### Data analysis

Data are presented as mean ± standard error of the mean (SEM) and number of cells is shown as N. Statistical comparison were evaluated by paired t-test for electrophysiological studies, unpaired t-test for all other assays. The accepted level of significance for the tests was *P* < 0.05.

## Additional Information

**How to cite this article**: Keung, W. *et al.* Non-cell autonomous cues for enhanced functionality of human embryonic stem cell-derived cardiomyocytes via maturation of sarcolemmal and mitochondrial K_ATP_ channels. *Sci. Rep.* **6**, 34154; doi: 10.1038/srep34154 (2016).

## Supplementary Material

Supplementary Information

## Figures and Tables

**Table 1:** Summary of action potential parameters of hESC-**V**CMs under different treatment.

Treatment	Action potential amplitude (mV)	Upstroke velocity (mV/ms)	Decay velocity (mV/ms)	Maximal diastolic potential (mV)
Control (N = 88)	87.56 ± 1.45	5.73 ± 0.67	−0.54 ± 0.03	−68.62 ± 0.81
Early CN (N = 74)	81.99 ± 1.63	5.47 ± 0.83	−0.68 ± 0.05	−67.68 ± 0.94
Late CN (N = 74)	81.27 ± 1.88	5.82 ± 1.09	−0.73 ± 0.05	−67.76 ± 1.08
CN+GLI (N = 25)	87.64 ± 3.22	5.22 ± 1.84	−0.63 ± 0.08	−71.66 ± 1.74
CN+HMR (N = 18)	85.86 ± 3.66	5.84 ± 1.76	−0.35 ± 0.04	−67.37 ± 1.64
Early P (N = 14)	90.01 ± 3.32	10.80 ± 2.64	−0.76 ± 0.09	−71.99 ± 2.30
Late P (N = 14)	92.95 ± 3.38	13.14 ± 3.30	−0.85 ± 0.13	−74.27 ± 2.34
P+HMR (N = 14)	91.74 ± 4.00	11.07 ± 2.91	−0.70 ± 0.11	−72.77 ± 2.25
CN+5HD (N = 13)	74.17 ± 3.63	7.57 ± 1.63	−0.58 ± 0.13	−59.78 ± 2.16
DIA (N = 18)	87.33 ± 3.17	3.59 ± 1.12	−0.67 ± 0.06	−70.73 ± 1.57

CN: cyanide (2 mM); GLI: glibenclamide (10 μM); HMR: HMR-1098 (100 μM); P: P-1075 (100 μM); 5HD: 5-hydroxydecanoate (100 μM); DIA: diazoxide (100 μM).

Early CN (or P) defined as the treatment at the beginning (2–3 min). Late CN (or P) defined as steady-state after the treatment.
