# Supplementary material for: Non-cell autonomous cues for enhanced functionality of human embryonic stem cell-derived cardiomyocytes via maturation of sarcolemmal and mitochondrial KATP channels
Source: Sci Rep. 2016 Sep 28;6:34154. doi: 10.1038/srep34154 (PMC5039730; doi:10.1038/srep34154)
Supplement: Supplementary Information [file srep34154-s1.pdf]

Non-cell autonomous cues for enhanced functionality of human embryonic stem cell-derived cardiomyocytes via maturation of sarcolemmal and mitochondrial K_{ATP} channels

Wendy Keung [1,2,+], Lihuan Ren[1,2,+], Sen Li[1,2], Andy On-Tik Wong [1,2], Anant Chopra [3,4], Chi-Wing Kong[1,2], Gordon F. Tomaselli [5], Christopher S. Chen [3,4], Ronald A. Li [1,2,6 *]

[1]Stem Cell & Regenerative Medicine Consortium, LKS Faculty of Medicine, University of Hong Kong, Hong Kong;
[2]Department of Physiology, University of Hong Kong, Hong Kong;
[3]Department of Bioengineering, Boston University, Boston, MA, USA;
[4]Harvard Wyss Institute for Biologically Inspired Engineering, Boston, MA, USA;
[5]Division of Cardiology, Department of Medicine, Johns Hopkins University, Baltimore, M.D., United States of America;
[6]Center of Cardiovascular Research, Icahn School of Medicine at Mount Sinai, New York, N.Y., United States of America

*Corresponding author
Ronald A. Li, Ph.D.
Stem Cell & Regenerative Medicine Consortium
The University of Hong Kong
L5 Hong Kong Jockey Club Building for Interdisciplinary Research
5 Sassoon Road, Pokfulam
Hong Kong
Tel: +852 28315388
Fax: +852 30175508
Email: ronaldli@hku.hk

[+]These authors contributed equally to this work

Supplemental Figure 1.

Role of mitochondrial $I_{K, ATP}$ activity in hESC-VCMs.

The effects of mitochondrial $I_{K, ATP}$ opener and blocker on cell survival after hypoxia. TUNEL assay of control (normoxia), hypoxia-treated hESC-VCMs in the presence of DNP (100 and 1000 μM) or diazoxide (10 - 1000 μM) with or without mitoK_{ATP} channel specific inhibitor 5HD (100 μM) as indicated. N = 20.

Supplemental Figure 2.

Effects of T3 treatment on action potential in quiescent HES2-VCMs. Representative tracings of action potentials (**A**) and action potential parameters comparison (**B**) of quiescent HES2-VCMs treated with or without T3 (100 nM) for 2 or 7 days. N = 21, 14 and 15 for control, T3 treatment for 2 and 7 days.

Supplemental Figure 3.

Effect of T3 treatment on cardioprotective effect of SarcKATP channels. TUNEL assay of normoxia and hypoxia (1% O_2)-treated hESC-VCMs and 300 nM T3-treated hESC-VCMs**,** in the presence of P1075 (100 μM) with or without HMR1098 (100 μM) as indicated. N = 8. *P< 0.05.

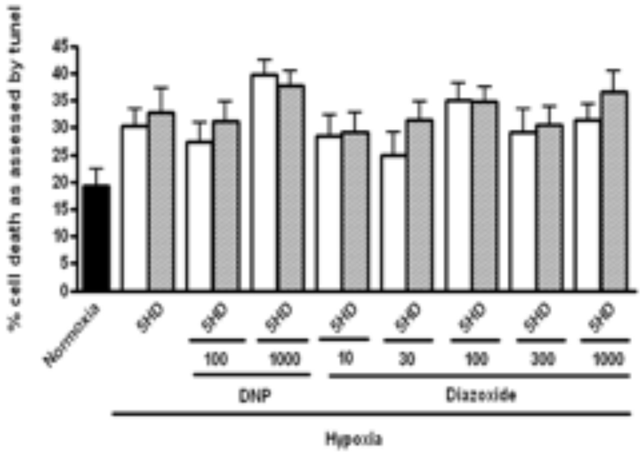

Supplemental figure 1

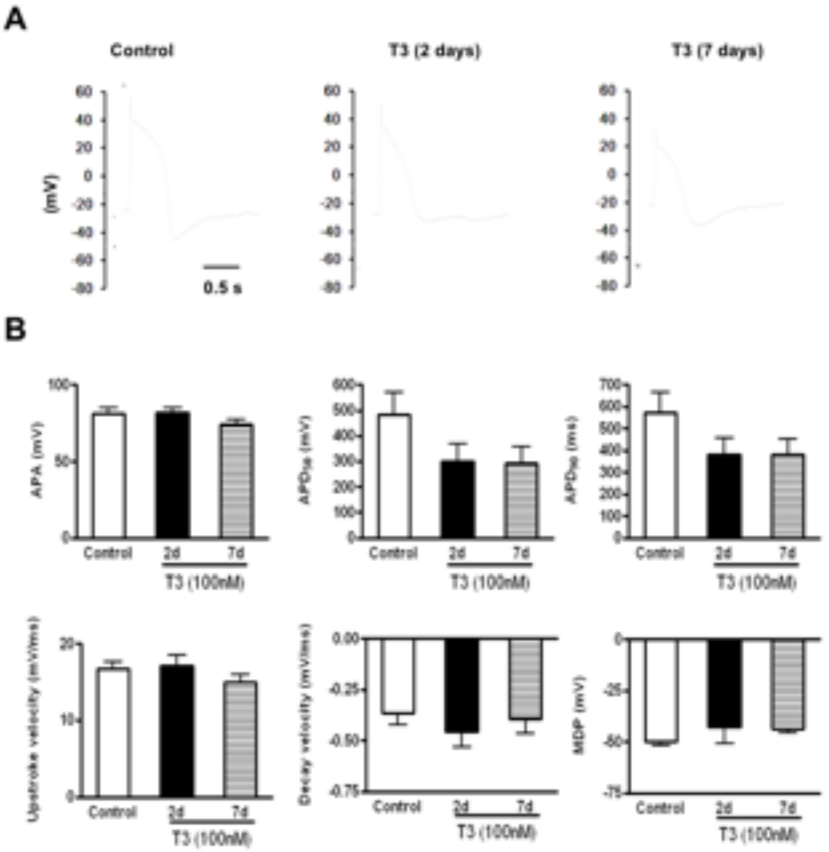

Supplemental figure 2

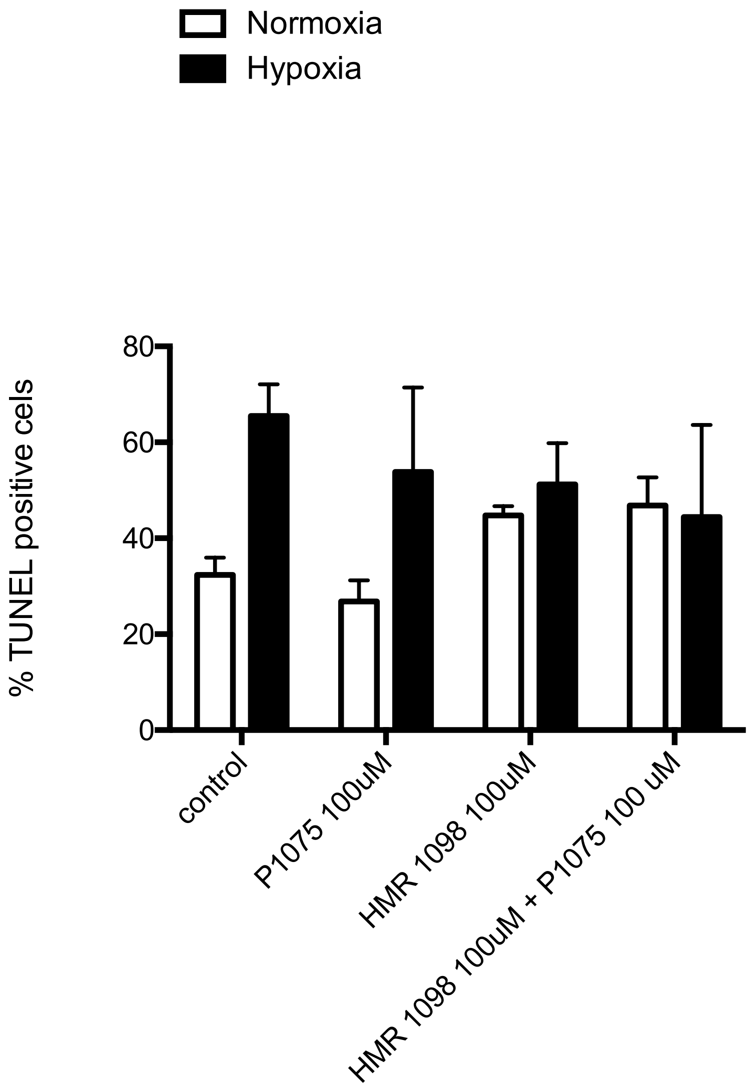

Supplemental Figure 3
